# Genetic influence of Apolipoprotein E gene ε2/ε3/ε4 isoforms on odds of mesial temporal lobe epilepsy

**DOI:** 10.4314/ahs.v21i2.48

**Published:** 2021-06

**Authors:** Tao Xu, Hui Zhang, Xueliang Qiu, Yuping Meng

**Affiliations:** 1 First Department of Neurology, The First Hospital of Zibo, Zibo City, Shandong Province, P.R. China; 2 The 960th Hospital of the Joint Logistics Support Force of the Chinese People's Liberation Army, Zibo City, Shandong Province, P.R. China; 3 Department of Neurology, Zibo Central Hospital, Zibo City, Shandong Province, P.R. China; 4 Blood purification center, The First Hospital of Zibo, Zibo City, Shandong Province, P.R. China

**Keywords:** Epilepsy, ApoE, isoforms, susceptibility

## Abstract

**Objective:**

The potential correlation between the ε2/ε3/ε4 variants of the ApoE (Apolipoprotein E) gene and the odds of mesial temporal lobe epilepsy was investigated.

**Methods:**

The database searching for eligible studies was performed in October 2020. A series of pooling analyses were conducted.

**Results:**

We enrolled a total of twelve case-control studies for pooling. Within the pooling analysis of ε4, there was an increased risk of mesial temporal lobe epilepsy in cases under the models of carrier ε4 vs. ε3, ε3ε4 vs. ε3ε3, and ε3ε4+ε4ε4 vs. ε3ε3 [P < 0.05, odds ratio (OR) > 1], compared with controls. Moreover, we observed similar positive results in the subgroup analyses of “China” and “Population-based control” under the genetic models of ε4 (P < 0.05, OR > 1). Nevertheless, we did not detect the significant difference between the mesial temporal lobe epilepsy cases and controls in the pooling analyses of ε2 (all P > 0.05).

**Conclusion:**

The ε3ε4 genotype of ApoE seems to be linked to the risk of mesial temporal lobe epilepsy for patients in China. More sample sizes are required to confirm the potential role of ApoE isoforms in the susceptibility to diverse types of epilepsy from different origins.

## Introduction

Epilepsy is a disease of the nervous system with disabling neurologic conditions, characterized by at least two unprovoked seizures more than twenty-four hours apart^1^–^4^. As the most common form of partial epilepsy with focal seizures, TLE (temporal lobe epilepsy) is characterized by recurrent, unprovoked focal seizures in the temporal lobe of the brain^5^–^7^. The MTLE (mesial temporal lobe epilepsy) is a highly prevalent indication for the surgical treatment^5^, ^8^. The pathophysiological mechanism of TLE or MTLE remains elusive. A growing number of genes and the relevant genetic variants are reportedly associated with the odds of clinical epilepsy disease, which contribute to the therapeutic advice during the personalized medicine^1^, ^9^.

Human ApoE (Apolipoprotein E) protein, encoded by the ApoE gene on chromosome 19, contains three protein isoforms (E2, E3, and E4) and is related to the transformation and metabolism of lipoproteins^10^–^12^. There are three common allelic forms of the human ApoE gene (ε2, ε4, and ε3), and six genotypes, namely ε3ε3, ε3ε2, ε2ε2, ε3ε4, ε4ε4, and ε2ε4, are generated by the combination of two different polymorphisms rs429358 and rs7412^12^–^15^. Several meta-analyses reported the statistical genetic relationship between the ApoE ε4 carrier and the risk of PD (Parkinson disease)^13^ or FTLD (frontotemporal lobar degeneration)^14^. Herein, we are interested in investigating whether ε2/ε3/ε4 isoforms of the ApoE gene is associated with the odds of TLE/MTLE, based on the available evidence^16^–^27^.

In the present study, we pooled the data of twelve eligible case-control studies to analyze the genetic correlation between ApoE ε2/ε3/ε4 isoforms and the susceptibility to the mesial temporal lobe epilepsy.

## Materials and methods

### Study identification

We tried to retrieve four databases, including PubMed, Embase (Excerpta medica database), Wanfang, CNKI (china national knowledge infrastructure), for the identification of relevant case-control studies, until October 2020. The searching terms were shown in Table S1.

### Screening criteria

Then, we excluded the records using the following criteria: (1) duplicate studies; (2) case report, meta-analysis, or review article; (3) meeting abstract or animal data; (4), not ApoE isoforms, or not TLE/MTLE data; (5) without full genotype of genotypic or allelic frequency data. We tried to send emails to the authors for the missing data. The included studies should contain the distribution data of ε2, ε3, ε4 allele, or the genotype frequencies of “ε2/ε2”, “ε2/ε3”, “ε2/ε4”, “ε3/ε3”, “ε3/ε4”, “ε4/ε4” in both TLE/MTLE cases and negative controls. Besides, after the assessment of the NOS (Newcastle-Ottawa Scale) system, only the studies with high quality (NOS score >=5) were included.

### Pooling analysis

We extracted the basic information of the first author, publication year, country, ethnicity, genotype frequency, control source, genotyping assay, and sample size in each study. Then, we performed a series of pooling analyses under the genetic models of allelic ε4 vs. total (ε3+ε2+ε4), allelic ε4 vs. ε3, allelic ε2 vs. total (ε3+ε2+ε4), allelic ε2 vs. ε3, carrier ε4 vs. total, carrier ε4 vs. ε3, carrier ε2 vs. total, carrier ε2 vs. ε3, ε4ε4 vs. ε3ε3 (homozygote), ε3ε4 vs. ε3ε3 (heterozygote), ε2ε2 vs. ε3ε3 (homozygote), ε3ε2 vs. ε3ε3 (heterozygote), ε3ε4+ε4ε4 vs. ε3ε3 (dominant), ε4ε4 vs. ε3ε3+ε3ε4 (recessive), ε3ε2+ε2ε2 vs. ε3ε3 (dominant), and ε2ε2 vs. ε3ε3+ε3ε2 (recessive). After pooling analysis of at least three case-control studies, we obtained the PA (P-value of the association test) and the value of the OR (95% CI) [odds ratio (95 % confidence interval)].

For the heterogeneity test, we obtained the PH (P-value of Cochran's Q statistic) and I2 value. When PH < 0.05 or I2 > 50%, the heterogeneity between studies was considered, and a random-effect model was applied for the DerSimonian and Laird statistics. If not, a fixed-effect model was for the Mantel-Haenszel statistics. Additionally, the subgroup analyses stratified by control source and country were performed.

### Sensitivity and publication bias

To assess the statistical stability of our pooling results, we performed a group of sensitivity analyses, in which each study was excluded sequentially. Besides, we employed both the Begg's test and Egger's test to evaluate publication bias. The presence of potential publication bias was considered when the P-value of Begg's /Egger's test (PB / PE) was larger than 0.05. Stata software (Stata Corporation, College Station, USA) was applied for the above analysis.

## Results

### Study inclusion

**Figure 1:**
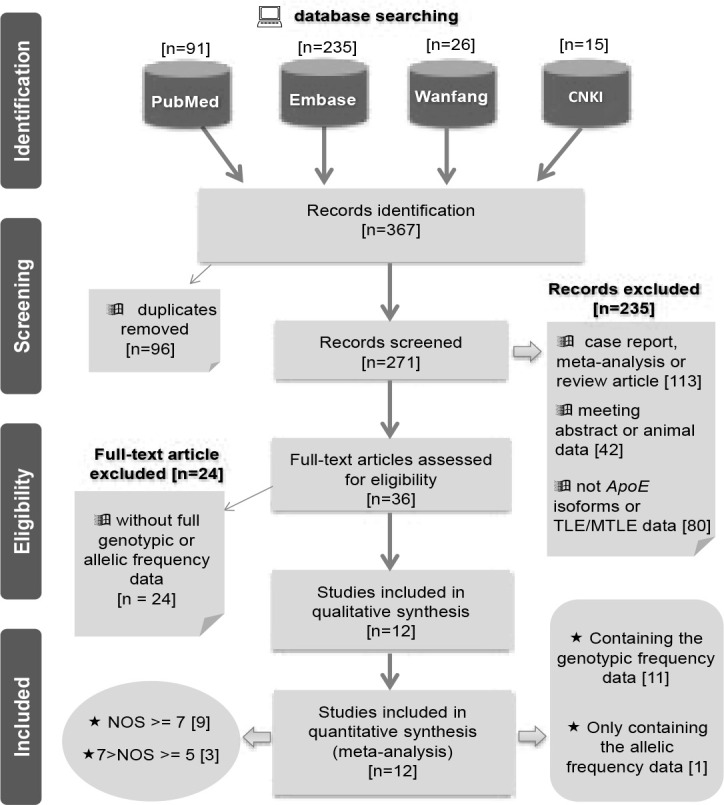
Flow chart for study identification.

As indicated in Figure 1, we obtained 91 records from PubMed, 235 records from the Embase, 26 records from the Wanfang, 15 records from the CNKI database. Then, based on our exclusion criteria, we excluded the 96 duplicates and other 235 unsuitable records. In total, 36 full-text articles were evaluated for eligibility. We then removed 24 articles because of “without full genotypic or allelic frequency data”. Finally, twelve eligible case-control studies16–27 with high-quality (NOS score >=5) were included. Of them, NOS scores of nine studies were larger than seven. We listed the basic information in Table 1. It should be noted that only the data of allelic frequency of ε3/ε2/ε4 was extracted from one study16, which was only used for the pooling analysis under the allelic model.

**Table 1:** Basic information data

First author, Year	Country	Ethnicity	ε2ε2/ε2ε3/ε2ε4/ ε3ε3/ε3ε4/ε4ε4	Disease	ε2ε2/ε2ε3/ε2ε4/ ε3ε3/ε3ε4/ε4ε4	Control Source	genotyping assay	NOS
Cavalleri, 2005	UK	Caucasian	230/20/36*	TLE	469/57/108*	PB	gene sequencing	6
Fu, 2010	China	Asian	6/91/9/358/88/8	TLE	8/106/6/344/91/3	PB	PCR-RFLP	8
Gambardella, 2005	Italy	Caucasian	0/13/2/101/21/1	TLE	1/38/3/227/27/1	PB	one-stage PCR	8
Gambardella, 1999	Italy	Caucasian	0/8/0/50/5/0	TLE	1/31/2/166/19/1	PB	PCR-RFLP	8
Huang, 2015	China	Asian	3/2/0/27/13/1	MTLE	0/3/0/13/3/0	HB	PCR-RFLP	5
Kumar, 2006	India	Asian	0/1/0/46/9/2	TLE	0/3/0/46/7/1	PB	PCR-RFLP	8
Leal, 2017	Portugal	Caucasian	0/15/3/133/37/0	MTLE	0/40/3/248/50/1	PB	PCR-RFLP	7
Li, 2007	China	Asian	1/12/0/64/17/0	MTLE	0/11/1/78/12/0	PB	gene sequencing	7
Li, 2016	China	Asian	3/39/2/209/55/0	MTLE	1/33/2/230/36/0	PB	gene sequencing	7
Salzmann, 2008	France	Caucasian	0/9/1/72/27/0	MTLE	0/25/5/151/43/3	PB	PCR-RFLP	7
Song, 2016	China	Asian	0/8/51/0/10/0	TLE	0/15/12/0/18/3	PB	gene sequencing	8
Yeni, 2005	Turkey	Asian	5/4/1/30/6/1	MTLE	10/13/0/30/4/5	HB	PCR-RFLP	6

TLE, temporal lobe epilepsy; MTLE, mesial temporal lobe epilepsy; PB, population-based; HB, hospital-based; PCR, polymerase chain reaction; RFLP, restriction fragment length polymorphism; NOS: Newcastle-Ottawa Scale

* the allelic frequency of ε3 /ε2 /ε4.

### Meta-analysis data of ε4

**Figure 2:**
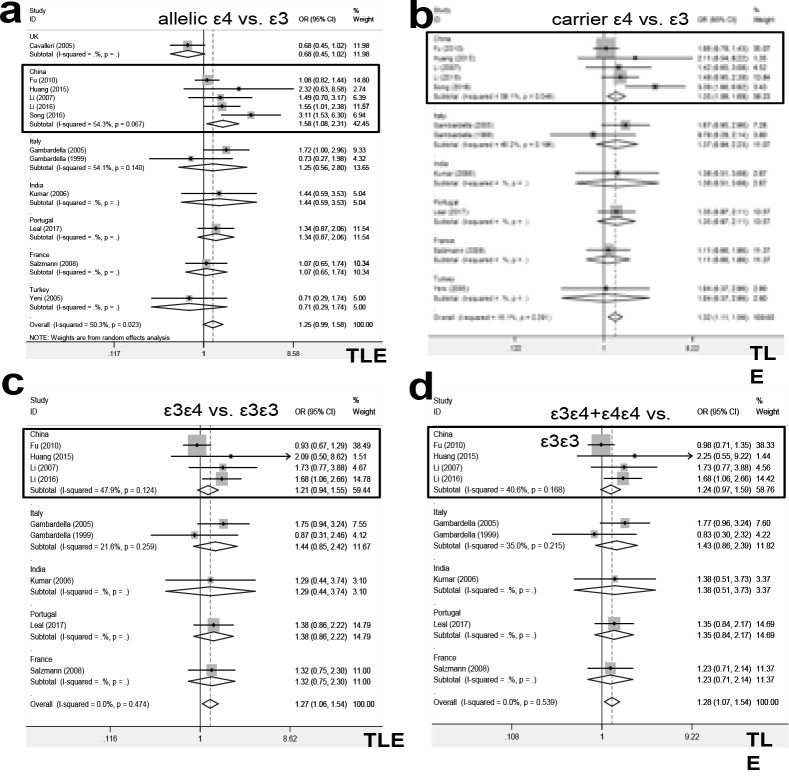
Subgroup analysis of TLE by country under the models of ε4. (a) allelic ε4 vs. ε3; (b) carrier ε4 vs. ε3; (c) ε3ε4 vs. ε3ε3; (d) ε3ε4+ε4ε4 vs. ε3ε3. The data of the “China” subgroup was marked with a rectangle.

As shown in Table S2, there were a total of twelve studies (1,823 cases and 2,551 controls) in the pooling analysis of TLE under the models of allelic ε4 vs. total and allelic ε4 vs. ε3. No significant statistical difference between the TLE patients and negative controls was detected (Table S2, PA >0.05). For the meta-analysis under the carrier ε4 vs. total and carrier ε4 vs. ε3 models (Table 2), eleven studies with 1,680 cases and 2,234 controls were enrolled. We observed an increased risk of TLE in cases, compared with controls, under the genetic models of carrier ε4 vs. total (Table 2, PA = 0.009, OR=1.24), carrier ε4 vs. ε3 (Table 2, PA = 0.001, OR=1.32), ε3ε4 vs. ε3ε3 (Table 3, PA = 0.011, OR=1.27), ε3ε4+ε4ε4 vs. ε3ε3 (Table S3, PA = 0.008, OR=1.28). These suggested that the ε3ε4 genotype of the ApoE gene was likely to be linked to the odds of TLE. Two factors of control source (population-based, PB), country (China) were then applied in our subgroup analyses. As shown in Table 2, Table 3, Table S2, and Table S3, we observed similar significant statistical differences between TLE cases and controls in the subgroups of “TLE/PB” under the models of carrier ε4 vs. total, carrier ε4 vs. ε3, ε3ε4 vs. ε3ε3, ε3ε4+ε4ε4 vs. ε3ε3 (PA < 0.05, OR > 1). In the subgroup analysis of “TLE/China”, there is an increased risk of TLE in cases under the models of carrier ε4 vs. ε3 (Table 2, PA = 0.007, OR=1.35), allelic ε4 vs. total (Table S2, PA = 0.045, OR=1.23), and allelic ε4 vs. ε3 (Table S2, PA = 0.018, OR=1.58), compared with controls. The forest plots for the subgroup analyses of TLE by country were shown in Figure 2.

**Table 2:** Pooling data under the carrier model

Comparison	Group	study	Association test			case	control

			OR (95% CI)	*P*_A_	z		
carrier ε4 vs. total	TLE	11	1.24 (1.06, 1.47)	0.009	2.64	1,680	2,234
	TLE/PB	9	1.24(1.05, 1.46)	0.013	2.48	1,587	2,153
	TLE/China	5	1.22 (0.98, 1.51)	0.071	1.81	1,077	1,029
	MTLE	6	1.35 (1.06, 1.72)	0.015	2.44	792	1,054
	MTLE/PB	4	1.34(1.04, 1.73)	0.022	2.28	699	973
	MTLE/China	3	1.49 (1.03, 2.15)	0.033	2.13	448	423

carrier ε4 vs. ε3	TLE	11	1.32(1.11, 1.56)	0.001	3.23	1,680	2,234
	TLE/PB	9	1.31 (1.11, 1.56)	0.002	3.13	1,587	2,153
	TLE/China	5	1.35(1.08, 1.68)	0.007	2.68	1,077	1,029
	MTLE	6	1.34(1.05, 1.71)	0.017	2.39	792	1,054
	MTLE/PB	4	1.34(1.04, 1.72)	0.024	2.26	699	973
	MTLE/China	3	1.51(1.05, 2.18)	0.028	2.20	448	423

carrier ε2 vs. total	TLE	11	0.91(0.76, 1.09)	0.296	1.05	1,680	2,234
	TLE/PB	9	0.94(0.78, 1.12)	0.467	0.73	1,587	2,153
	TLE/China	5	1.03(0.83, 1.27)	0.803	0.25	1,077	1,029
	MTLE	6	0.88(0.68, 1.18)	0.425	0.80	792	1,054
	MTLE/PB	4	0.96(0.71, 1.29)	0.779	0.28	699	973
	MTLE/China	3	1.15(0.78, 1.71)	0.483	0.70	448	423

carrier ε2 vs. ε3	TLE	11	0.96 (0.71, 1.30)	0.776	0.28	1,680	2,234
	TLE/PB	9	1.02(0.74, 1.41)	0.906	0.12	1,587	2,153
	TLE/China	5	1.34(0.79, 2.26)	0.280	1.08	1,077	1,029
	MTLE	6	0.89 (0.67, 1.17)	0.392	0.86	792	1,054
	MTLE/PB	4	0.96(0.71, 1.29)	0.775	0.29	699	973
	MTLE/China	3	1.16(0.78, 1.73)	0.452	0.75	448	423

TLE, temporal lobe epilepsy; PB, population-based control; MTLE, mesial temporal lobe epilepsy; OR, odds ratio; CI, confidence interval; *P*_A_, *P*-value in association test.

**Table 3:** Pooling data under the homozygotic and heterozygotic models

Comparison	Group	study	Association test			case	control

			OR (95% CI)	*P*_A_	z		
ε4ε4 vs. ε3ε3	TLE	7	1.53(0.67, 3.47)	0.312	1.01	999	1,445
	TLE/PB	6	1.53(0.66, 3.58)	0.324	0.99	958	1,429
	MTLE	3	0.56(0.11, 3.06)	0.518	0.65	310	512

ε3ε4 vs. ε3ε3	TLE	9	1.27(1.06, 1.54)	0.011	2.54	1,344	1,801
	TLE/PB	8	1.26(1.04, 1.53)	0.016	2.41	1,303	1,785
	TLE/China	4	1.21(0.94, 1.55)	0.135	1.50	840	810
	MTLE	5	1.52(1.17, 1.97)	0.002	3.12	655	868
	MTLE/PB	4	1.50(1.15, 1.96)	0.003	1.02	614	852
	MTLE/China	3	1.73(1.18, 2.54)	0.006	2.76	386	372

ε2ε2 vs. ε3ε3	TLE	6	1.21(0.56, 2.63)	0.630	0.48	987	1,291
	TLE/PB	5	1.09(0.48, 2.46)	0.842	0.20	955	1,275
	TLE/China	4	1.26(0.55, 2.89)	0.585	0.55	815	827
	MTLE	3	3.42(0.70, 16.73)	0.129	1.52	360	369
	MTLE/China	3	3.42(0.70, 16.73)	0.129	1.52	360	369

ε3ε2 vs. ε3ε3	TLE	9	0.87(0.71, 1.07)	0.188	1.32	1,263	1,804
	TLE/PB	8	0.88 (0.72, 1.08)	0.231	1.20	1,231	1,788
	TLE/China	4	0.95(0.74, 1.22)	0.685	0.41	815	827
	MTLE	5	0.97(0.71, 1.33)	0.853	0.19	589	833
	MTLE/PB	4	1.00(0.73, 1.38)	0.243	0.01	557	817
	MTLE/China	3	1.22(0.80, 1.86)	0.362	0.91	360	369

TLE, temporal lobe epilepsy; PB, population-based control; MTLE, mesial temporal lobe epilepsy; OR, odds ratio; CI, confidence interval; *P*_A_, *P*-value in association test.

**Figure 3:**
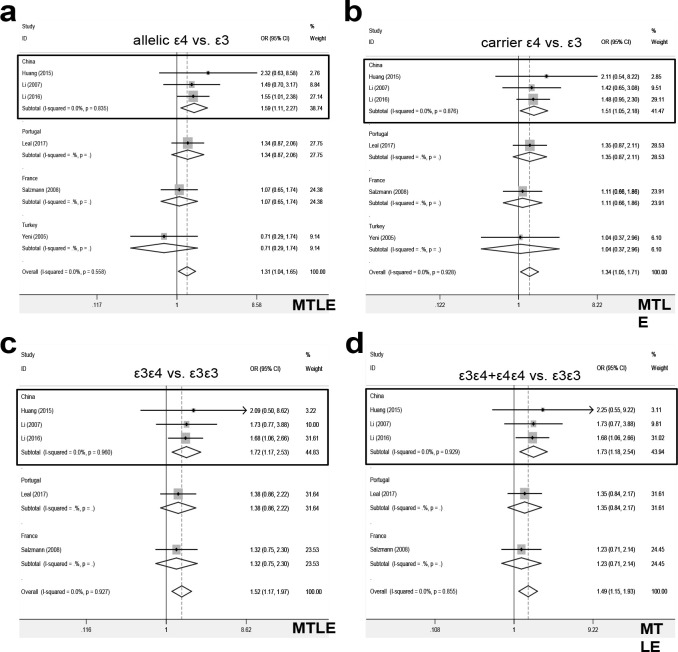
Subgroup analysis of MTLE by country under the models of ε4. (a) allelic ε4 vs. ε3; (b) carrier ε4 vs. ε3; (c) ε3ε4 vs. ε3ε3; (d) ε3ε4+ε4ε4 vs. ε3ε3. The data of the “China” subgroup was marked with a rectangle.

Further, we performed a series of pooling analyses of ε4, only including the data of MTLE cases. Compared with controls, there was an increased risk of MTLE in cases under the models of carrier ε4 vs. total (Table 2, PA = 0.015, OR =1.35), carrier ε4 vs. ε3 (Table 2, PA = 0.017, OR =1.34), ε3ε4 vs. ε3ε3 (Table 3, PA = 0.002, OR =1.52), allelic ε4 vs. total (Table S2, PA = 0.026, OR =1.29), allelic ε4 vs. ε3 (Table S2, PA = 0.020, OR =1.31), ε3ε4+ε4ε4 vs. ε3ε3 (Table S3, PA = 0.003, OR =1.49). Also, we observed similar positive conclusions in the subgroup analysis of “MTLE/PB” and “MTLE/China” (Table 2-3, Table S2-S3, all PA <0.05, OR >1). The forest plots for the subgroup analyses of MTLE by country were shown in Figure 3. Thus, ε3ε4 genotype is more likely to be associated with the susceptibility of Chinese patients to the mesial temporal lobe epilepsy.

### Meta-analysis data of ε2

For the pooling analysis of ε2, we did not detect a significant difference between the TLE/MTLE cases and negative controls under the models of carrier ε2 vs. total, carrier ε2 vs. ε3, ε2ε2 vs. ε3ε3, ε3ε2 vs. ε3ε3, allelic ε2 vs. total, allelic ε2 vs. ε3, ε3ε2+ε2ε2 vs. ε3ε3, ε2ε2 vs. ε3ε3+ε3ε2 (Table 2–3, Table S2-S3, all PA > 0.05). Also, no positive conclusions were observed in the subgroup analyses by the control source or country under any genetic model of ε2 (Table 2–3, Table S2-S3, all PA > 0.05). The forest plots for the subgroup analyses by country were shown in Figure S1-S2. These suggested that ε2 allele, or ε3ε2, ε2ε2 genotype may not be strongly linked to the odds of TLE or MTLE.

### Heterogeneity analysis

As shown in Table S4, we utilized a random-effect model (DerSimonian and Laird statistics) for the association test under the genetic models of carrier ε2 vs. ε3 (PH < 0.021, I2 = 52.6%), allelic ε4 vs. ε3 (PH = 0.023, I2 = 50.3%), and allelic ε2 vs. ε3 (PH = 0.009, I2 = 56.4%), respectively. And a fixed-effect model (Mantel-Haenszel statistics) was applied for others, due to the lack of between-study heterogeneity (Table 4, PH > 0.05 and I2 < 50.0 %).

### Sensitivity and publication bias

Our results of sensitivity analysis indicated the statistical stability of the above conclusions. We showed the data of the carrier models (carrier ε4 vs. total; carrier ε4 vs. ε3; carrier ε2 vs. total; carrier ε2 vs. ε3.) as examples in Figure S3. As shown in Table S4, we did not observe significant publication bias in all comparisons (PB>0.05, PE>0.05). Figure S4 presents the publication bias plots in Egger's test under the carrier models (carrier ε4 vs. total; carrier ε4 vs. ε3; carrier ε2 vs. total; carrier ε2 vs. ε3) as examples.

## Discussion

No statistical differences in ApoE ε 4 allelic frequencies between MTLE-HS (mesial temporal lobe epilepsy with hippocampal sclerosis) cases and patients and healthy controls were detected; ApoEε 4 carriers may be related to earlier MTLE-HS onset in Portugal^22^. ApoE ε 4 allele was reportedly associated with the odds of Chinese NLMTLE (nonlesional mesial temporal lobe epilepsy)^23^, and TLE with prior trauma^17^. Nevertheless, the ApoEε 4 allele was reportedly unrelated to the onset age of epilepsy, duration, or the silent period in the refractory TLE group^17^. Also, no genetic correlation between ApoEε 4 isoform and the onset age or outcome after surgery of MTLE-HS was observed in Turkey^27^. The lack of the genetic role of ApoE isoform in the occurrence of nonlesional TLE cases in Italy was reported^19^. Thus, this issue merits the preformation of a meta-analysis.

There were eight studies included in a relevant meta-analysis of Kauffman, M. A. et al. in 2010, which evaluated the effect of ApoEε 4 isoform on the age at onset of temporal lobe epilepsy28. In 2019, another meta-analysis containing nine studies reported that ApoE ε 4 isoform is associated with a high susceptibility to Asian epilepsy cases^29^. In the present study, we enrolled the available eligible studies and used the different analysis strategies to explore the genetic role of the allelic and genotypic frequencies of ApoE ε2/ε3/ε4 isoforms in the risk of TLE or MTLE. After the database searching, we enrolled a total of twelve eligible case-control studies for the pooling analysis under a series of genetic models, namely allelic ε4 vs. total, allelic ε4 vs. ε3, allelic ε2 vs. total, allelic ε2 vs. ε3, carrier ε4 vs. total, carrier ε4 vs. ε3, carrier ε2 vs. total, carrier ε2 vs. ε3, ε4ε4vs. ε3ε3, ε3ε4 vs. ε3ε3, ε2ε2 vs. ε3ε3, ε3ε2 vs. ε3ε3, ε3ε4+ε4ε4 vs. ε3ε3, ε4ε4 vs. ε3ε3+ε3ε4, ε3ε2+ε2ε2 vs. ε3ε3, and ε2ε2 vs. ε3ε3+ε3ε2. Our findings revealed that the ε3ε4 genotype of the ApoE gene is more likely to be linked to the odds of mesial temporal lobe epilepsy cases in China, which was considered statistically credible by the preformation of sensitivity analyses.

Despite this, we should consider the findings of our pooling analyses with precaution. There are insufficient cases and controls in some comparisons. For instance, even though we observed a statistical association between the ε3ε4 genotype of ApoE and an increased MTLE susceptibility for Chinese cases, only three case-control studies^20^, ^23^, ^24^ were included for the pooling analysis. Although the lack of more considerable publication bias in all comparisons, less than ten case-control studies were included for the pooling analysis under the models of ε4ε4 vs. ε3ε3, ε3ε4 vs. ε3ε3, ε2ε2 vs. ε3ε3, ε3ε2 vs. ε3ε3, ε3ε4+ε4ε4 vs. ε3ε3, ε4ε4 vs. ε3ε3+ε3ε4, ε3ε2+ε2ε2 vs. ε3ε3, ε2ε2 vs. ε3ε3+ε3ε2. We observed a high level of between-study heterogeneity under the genetic models of carrier ε2 vs. ε3, allelic ε4 vs. ε3, and allelic ε2 vs. ε3.

Besides, the potential effect of non-ε2/ε3/ε4 ApoE isoforms or the combined impact of ApoE isoforms with other variants, [e.g., ABCA7 (ATP Binding Cassette Subfamily A Member 7) rs4147929 or CD33 rs3865444, etc.], on the odds of TLE/MTLE should be considered when the more sample sizes were available. In addition, temporal lobe epilepsy is often accompanied by some other neurological pathologies, such as hippocampal sclerosis^27^, ^30^. The factors of clinical features should be fully considered for the adjusted estimation in the future as well.

## Conclusion

Taken together, our data suggested that the ε3ε4 genotype of the ApoE gene may be related to enhanced susceptibility to mesial temporal lobe epilepsy for patients in China. Large-scale publications are required to verify the role of more ApoE variants in the risk of cases with different types of epilepsy in other regions.
